# Nitric Oxide Orchestrates a Power-Law Modulation of Sympathetic Firing Behaviors in Neonatal Rat Spinal Cords

**DOI:** 10.3389/fphys.2018.00163

**Published:** 2018-03-06

**Authors:** Chun-Kuei Su, Yi-Yin Chen, Chiu-Ming Ho

**Affiliations:** ^1^Institute of Biomedical Sciences, Academia Sinica, Taipei, Taiwan; ^2^Department of Anesthesiology, Taipei Veterans General Hospital and National Yang-Ming University, Taipei, Taiwan

**Keywords:** autonomic nervous system, heterogeneity, nicotinic receptor, nitric oxide synthase, power function, sympathetic preganglionic neuron

## Abstract

Nitric oxide (NO) is a diffusible gas and has multifarious effects on both pre- and postsynaptic events. As a consequence of complex excitatory and inhibitory integrations, NO effects on neuronal activities are heterogeneous. Using *in vitro* preparations of neonatal rats that retain the splanchnic sympathetic nerves and the thoracic spinal cord as an experimental model, we report here that either enhancement or attenuation of NO production in the neonatal rat spinal cords could increase, decrease, or not change the spontaneous firing behaviors recorded from splanchnic sympathetic single fibers. To elucidate the mathematical features of NO-mediated heterogeneous responses, the ratios of changes in firing were plotted against their original firing rates. In log-log plots, a linear data distribution demonstrated that NO-mediated heterogeneity in sympathetic firing responses was well described by a power function. Selective antagonists were applied to test if glycinergic, GABAergic, glutamatergic, and cholinergic neurotransmission in the spinal cord are involved in NO-mediated power-law firing modulations (plFM). NO-mediated plFM diminished in the presence of mecamylamine (an open-channel blocker of nicotinic cholinergic receptors), indicating that endogenous nicotinic receptor activities were essential for plFM. Applications of strychnine (a glycine receptor blocker), gabazine (a GABA_A_ receptor blocker), or kynurenate (a broad-spectrum ionotropic glutamate receptor blocker) also caused plFM. However, strychnine- or kynurenate-induced plFM was diminished by L-NAME (an NO synthase inhibitor) pretreatments, indicating that the involvements of glycine or ionotropic glutamate receptor activities in plFM were secondary to NO signaling. To recapitulate the arithmetic natures of the plFM, the plFM were simulated by firing changes in two components: a step increment and a fractional reduction of their basal firing activities. Ionotropic glutamate receptor activities were found to participate in plFM by both components. In contrast, GABA_A_ receptor activities are involved in the component of fractional reduction only. These findings suggest that NO orchestrates a repertoire of excitatory and inhibitory neurotransmissions, incurs a shunting effect on postsynaptic membrane properties, and thus, alters sympathetic firing in a manner of plFM. We propose that the plFM mediated by NO forms a basic scheme of differential controls for heterogeneous sympathetic regulation of visceral functions.

## Introduction

Heterogeneity in neurons and complexity of their wiring are the major challenges in studying a neural network. While a given test to perturb an operating neural circuit often yields discrepant responses, this common phenomenon is generally considered as the consequences of random variations. The mechanisms underlying the variations that could detail the neural heterogeneity and its complex responses were mostly ignored and nearly unexplored.

**Figure 1:**
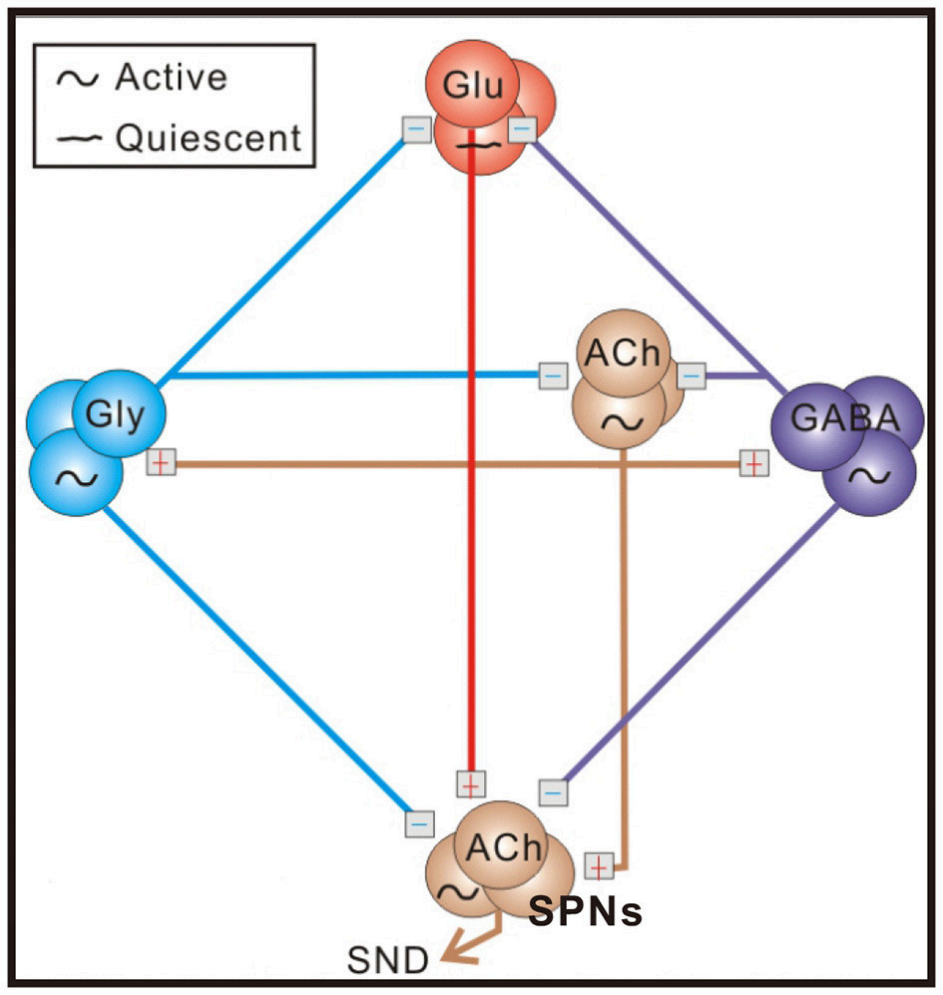
A schematic diagram of the sympathetic-correlated neural circuit in the spinal cord. ACh, GABA, Gly, and Glu label cholinergic, GABAergic, glycinergic, and glutamatergic neurons, respectively. Under control conditions, sympathetic preganglionic neurons (SPNs) generating sympathetic nerve discharges (SND) are activated by cholinergic inputs and inhibited by GABAergic and glycinergic inputs. Blockades of endogenous GABA_A_ and glycine receptor activities enhance excitatory cholinergic inputs and transform tonic SND into bursts by unleashing an ionotropic glutamate receptor-mediated input. Symbols of active and quiescent indicate firing properties under control conditions. See text for references of supporting evidence.

Neonatal rat spinal cords contain sufficient neural elements for spontaneous generation of sympathetic nerve discharges (SND; Su, 1999; Pierce et al., 2010). As illustrated in Figure 1, operations of the sympathetic-correlated neural circuit require endogenously active cholinergic neurotransmission working via nicotinic receptors, which subsequently activates GABAergic and glycinergic spinal interneurons (Chen and Su, 2006). These inhibitory neurons silence the glutamatergic neurons that may transform tonic SND into bursts (Su, 2001; Su et al., 2003). The observations of the spinally-originated SND are not limited to rats in neonatal stages. It has also been observed in adult rats or cats that have their neuraxis transected at the cervical spinal cord (Ardell et al., 1982; Osborn et al., 1987; Hong et al., 1994) or in a perfused preparation obtained from adult mouse spinal cords (Chizh et al., 1998).

Complexity in the operation of the sympathetic-correlated neural circuit in the spinal cord is clearly revealed by the heterogeneous responses of their efferent fiber activities, when a variety of antagonists were applied to alter the endogenous receptor activities. Our previous studies demonstrate that GABAergic, glycinergic, and glutamatergic spinal neurons working, respectively, via GABA_A_ receptors, glycine receptors, and ionotropic glutamate receptors exert functionally distinct influences on different sympathetic fiber activities (Ho et al., 2013; Su, 2016). For those fibers of low and high spontaneous activities, their firing tends to be increased and decreased, respectively, by inhibition of endogenous GABA_A_ or glycine receptor activities. This discrepancy in change of firing is inversely related to individual fiber activities and well described by a power function of exponent <1 (Su, 2016). As the mathematical features of power law predicted, such a change in population firing behaviors elicited by blocking endogenous GABA_A_ or glycine receptor activities cannot be defined by a simple arithmetic mean for simply saying an increase or a decrease of population firing and is lacking a finite variance. While intriguing, the neural mechanisms underlying such a heterogeneous response that is characterized by the power-law firing modulations (plFM) remain elusive.

Nitric oxide (NO) is a diffusible gas exerting multifarious effects on both pre- and post-synaptic events to alter the synaptic efficacy and the neuronal excitability. NO is a product of L-arginine (Arg) catalyzed by nitric oxide synthases (NOS). Many sympathetic preganglionic neurons (SPNs) express NOS (Blottner and Baumgarten, 1992; Saito et al., 1994; Anderson et al., 1995; Doone et al., 1999). We hypothesize that NOS activities in SPNs, by amalgamating various synaptic events, play a pivotal role in heterogeneous modulation of sympathetic firing behaviors, which can be revealed as plFM.

Using oligofiber recording techniques, we simultaneously recorded several sympathetic single-fiber activities that were spontaneously generated from neonatal rat spinal cords (Su et al., 2013). We determined if manipulation of NO production would cause the plFM. We further explored the neural mechanisms underlying the NO-mediated plFM by pretreatments of various antagonists that interrupted glycine, GABA_A_, ionotropic glutamate, or nicotinic receptor activities. We attempted to explain NO-mediated plFM by a simple arithmetic simulation. Findings in this study strongly support an emerging notion that heterogeneous outcomes obtained from complex neural computations can simply be equated by a power function.

## Materials and methods

### Animals

Experiments were performed using neonatal Sprague-Dawley rats of age 1–6 postnatal days. All surgical and experimental procedures were approved by the Institutional Animal Care and Utilization Committee of Academia Sinica (Protocol#: RMiRaIBMSC2011081) in accordance with the Guide for the Care and Use of Laboratory Animals of the Agriculture Council of Taiwan.

### Splanchnic sympathetic nerve–thoracic spinal cord preparations *in vitro*

*En bloc* preparations retaining the splanchnic sympathetic nerve–thoracic spinal cord (T1–T12) were prepared following surgical procedures as previously described (Su, 1999; Ho et al., 2013). Briefly, neonatal rats were made unconscious by hypothermia (Danneman and Mandrell, 1997), followed by a prompt midcollicular decerebration. During dissection, the reduced preparation was immersed in ~4°C artificial cerebrospinal fluid (aCSF; in mM: 128 NaCl, 3 KCl, 1.5 CaCl_2_, 1.0 MgSO_4_, 24 NaHCO_3_, 0.5 NaH_2_PO_4_, 30 D-glucose, and 3 ascorbate; equilibrated with 95% O_2_-5% CO_2_). A stub of the splanchnic sympathetic nerves was freed from surrounding tissues and its distal end was severed adjacent to a ganglion termed as the suprarenal ganglion (Baljet and Drukker, 1979) or as the cardiac ganglion (Greene, 1963). The nerve stub comprises predominantly the sympathetic preganglionic fibers (Celler and Schramm, 1981; Sapru et al., 1982). The nerve-thoracic spinal cord preparation (T1–T12) was then immersed in a bath chamber containing freshly-oxygenated 30-ml aCSF with temperature maintained at 24.5 ± 1°C. Dissociation of the nerve bundles was performed by incubating the splanchnic nerves for ~90 min in a glass micropipette containing 0.5% collagenase (Type IV collagenase, C5138, Sigma-Aldrich, buffered by Hanks' Balanced Salt Solution, 14185-052, Invitrogen Corporation).

### Neural recordings

Borosilicate glass micropipettes (AM-system, 5928, Carlsborg, Washington) were tapered using a horizontal puller (P-97, Sutter Instrument, Novato, California) to make long-shank recording electrodes with tips ~5 μm in diameter and back-filled with aCSF. Electrical signals were pre-amplified (DAM50; World Precision Instruments, Sarasota, Florida), amplified (NL106, Digitimer Ltd., Hertfordshire, England), bandpass filtered at 10–3,000 Hz (NL126, Digitimer Ltd.), and stored on a pulse-code modulation tape recorder (Neuro-Corder DR-890; Cygnus Technology Inc., Delaware Water Gap, Pennsylvania). Analog signals were digitized in real time using a National Instrument-based data acquisition system (NI-PCI-6010, National Instrument, Austin, Texas) and processed using customized LabVIEW programs (version 15.0.1.1f2, National Instrument) incorporated with MATLAB scripts (version 8.5.0 The MathWorks, Inc., Natick, Massachusetts). To avoid aliasing and sampling jitter for precise waveform alignments at spike peaks, signals were first oversampled at 40 kHz and then downsampled to 10 kHz by interpolation algorithm to keep file size small. All signals were digitally corrected for amplification gains and expressed in units of μV for computational analyses.

### Acquisition of single-fiber activities

Automation of spike detection and sorting was primarily based on its waveform features as previously described (Su et al., 2013). Briefly, data clusters were automatically selected by *k*-means clustering algorithms followed by verification of the waveform homogeneity using principal component analysis to represent data and using Hotelling's T^2^ distances as criteria to purge those data located distant from the cluster centroids (i.e., T^2^-selection). The complex waveforms with large T^2^ distances that might result from spike overlapping were resolved using a subtraction algorithm (SA) to subtract an ideal spike waveform from the complex waveforms, followed by determining if the extracted waveforms truly occur during the recording. After partially resolving overlapped spikes by SA, both T^2^-selected and SA-retrieved spikes were combined as unit activities. A unit activity was verified by its probability distribution of interspike intervals (ISIs) and was considered to be truly originated from a single fiber if occurrence of the spikes did not violate a refractory period of 3-ms. Supplementary Figure 1 shows an example of spike sorting.

### Drugs and drug applications

Reagents purchased from Sigma-Aldrich included L-Arginine (Arg, NO precursor), L-argininamide dihydrochloride (an Arg derivative of effects similar to Arg; Ishii et al., 1991), eserine salicylate (Esr, acetylcholine esterase inhibitor), gabazine (Gab, competitive GABA_A_ receptor antagonist), kynurenic acid (Kyn, broad-spectrum ionotropic glutamate receptor blocker), mecamylamine hydrochloride (Meca, nicotinic receptor open-channel blocker), N_ω_-Nitro-L-arginine methyl ester hydrochloride (L-NAME, NOS inhibitor), (–)-nicotine hydrogen tartrate salt (Nict, nicotinic cholinergic receptor agonist), and strychnine hydrochloride (Stry, noncompetitive glycine receptor antagonist). All drugs were dissolved in water to prepare concentrated stock solutions. Drug concentrations were chosen to avoid an abolition of spontaneous firing activities. A final concentration of 50 μM Arg or L-argininamide, 10 μM Esr, 20 μM Gab, 400 μM Kyn, 1 μM Meca, 100 μM L-NAME, 1 μM Nict, and 2 μM Stry was achieved by adding an aliquot of the stock solutions directly to the bath chamber. Drug incubation times varied according to their responses. In applications of Gab, Kyn, Meca, or Stry, fast responses were often elicited and an elapse of 10 min after drug applications was allowed for equilibration. In applications of Arg or L-NAME, incubation times were extended to 25–40 min. Unless otherwise mentioned, the drug-induced responses were evaluated by comparing the firing activities that appeared in a 20-min epoch prior to drug applications with those in another 20-min epoch following the drug incubations.

### Data analysis

The number of spikes that occurred in a 20-min epoch was divided by 1,200 s to calculate the average firing rates (AFR). The AFR during drug applications divided by the AFR prior to drug applications were taken as AFR ratios. To detect if there was a trend that drug-induced changes in firing activities were related to their activities prior to drug applications, AFR ratios were plotted against their AFR prior to drug applications. In log-log plots, the data are usually scattered in a linear manner and thus, regressed by a power function using Origin (ver. 8.0725, OriginLab Corporation, Northampton, Massachusetts):

(1) $$\begin{array}{r} y=ax^{b} \end{array}$$

where *y* is the drug-induced change of AFR ratios, *a* is the intercept, *x* is AFR and the exponent *b* is the slope. To evaluate the uncertainty of the slope, the standard deviation of the slope (*s*_*b*_) was acquired by the regression using LINEST, a built-in-function of Microsoft Excel (2016), after log transforms of AFR and AFR ratios. The 95% confidence intervals of *b* (CI_*b*_) were calculated by the equation:

(2) $$\begin{array}{r} CI_{b}=b\pm t_{n-2}•s_{b} \end{array}$$

where *n* is the sample size and *t* a two-sided *t*-value of significance level at 0.025. In some experiments, two regressed lines acquired from the same drug tests in experiments with and without another drug pretreatment were compared. To verify if a drug pretreatment was effective in changing the patterns of data distribution, the data obtained from the experiments with and without the pretreatments were pooled and used to test if there existed a significant interaction between the groups, i.e., to test the significance of the product of a dummy variable “Group” (*G*) and AFR (Andrade and Estévez-Pérez, 2014). The pooled data were regressed by a multiple linear regression model using SPSS (ver.21, IBM Corporation, Armonk, New York):

(3) $$\begin{array}{r} \log\left(y_{p}\right)=c_{0}+c_{1}\log\left(x_{p}\right)+c_{2}G+c_{3}G\cdot \log\left(x_{p}\right) \end{array}$$

where *y*_*p*_ is the pooled AFR ratio from both groups, *c*_0−3_ are the coefficients, *x*_*p*_ is the pooled AFR, and *G* is the dummy variable. The presence of an interaction between *G* and log(*x*_*p*_) was indicated by the statistical significance of *c*_3_.

To recapitulate the physiological contexts of a data distribution in power-law manners, a simulation was implemented assuming that the drug-induced AFR changes were attributed to a step increment and a fractional reduction in firing. The rationale for including the component of the step increment to address the changes in firing is because a linear relationship often exists between the firing rates and the injected stimulus currents. Thus, a step change of firing may occur when the synaptic currents are altered. On the other hand, the rationale for including the component of the fractional reduction of the original firing is because a concomitant change in the membrane conductance often occurs along with the changes in receptor and ion channel activities. We tested if a change in passive membrane properties could contribute to a fractional component of firing changes. The arithmetic components were acquired by curve fitting using Origin. First, AFR after a treatment (*x*_*t*_) is expressed as a function of their original AFR prior to the treatment (*x*_*o*_):

(4) $$\begin{array}{r} x_{t}=c_{s}+\left(1-c_{f}\right)•x_{o} \end{array}$$

where *c*_*s*_ is the coefficient of step increment and *c*_*f*_ the coefficient of fractional reduction. A plot of the AFR ratios as calculated by $\frac{x_{t}}{x_{o}}$ against *x*_*o*_ mimics a data distribution in power-law manners. Second, the data obtained from drug-induced changes in firing were used to construct a plot of *x*_*t*_ against *x*_*o*_ and further fitted with the above equation to evaluate *c*_*s*_ and *c*_*f*_. Supplementary Figure 2 illustrates a synergistic contribution of these two arithmetic components to a data distribution in the power-law manners. Unless otherwise mentioned, values are expressed as mean ± SEM and a *P* < 0.05 is considered significant.

## Results

### Heterogeneity in NO modulation of sympathetic firing is described by a power function

**Figure 2:**
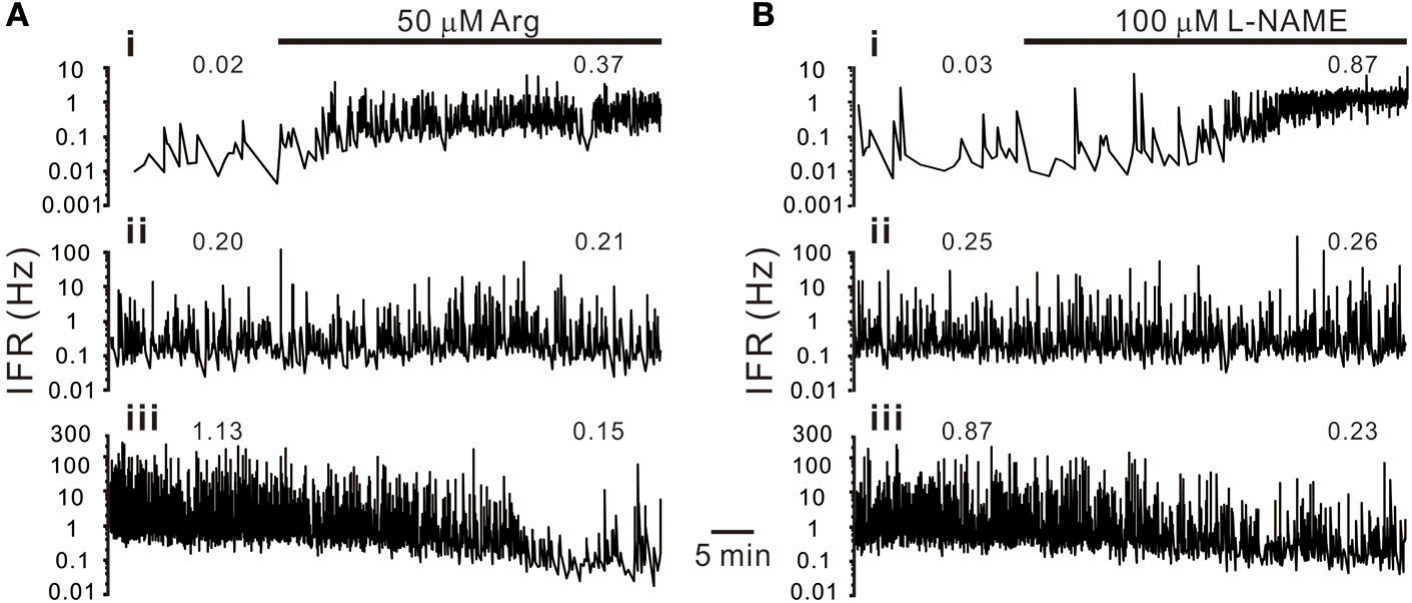
Heterogeneity in sympathetic firing responses elicited by bath applications of L-arginine (Arg, an NO precursor; **A**) or L-NAME (an NOS inhibitor; **B**). Traces show the time course of the splanchnic sympathetic single-fiber activities. IFR, instantaneous firing rates. Numerical values on top of traces are average firing rates (AFR). Traces i–iii show firing increased, not apparently changed or decreased. Examples show apparent differences in their basal activity levels under control conditions. Firing activities in the fibers of low basal activity were increased, and in those of high basal activity decreased, by either enhancement or diminution of NO production.

**Figure 3:**
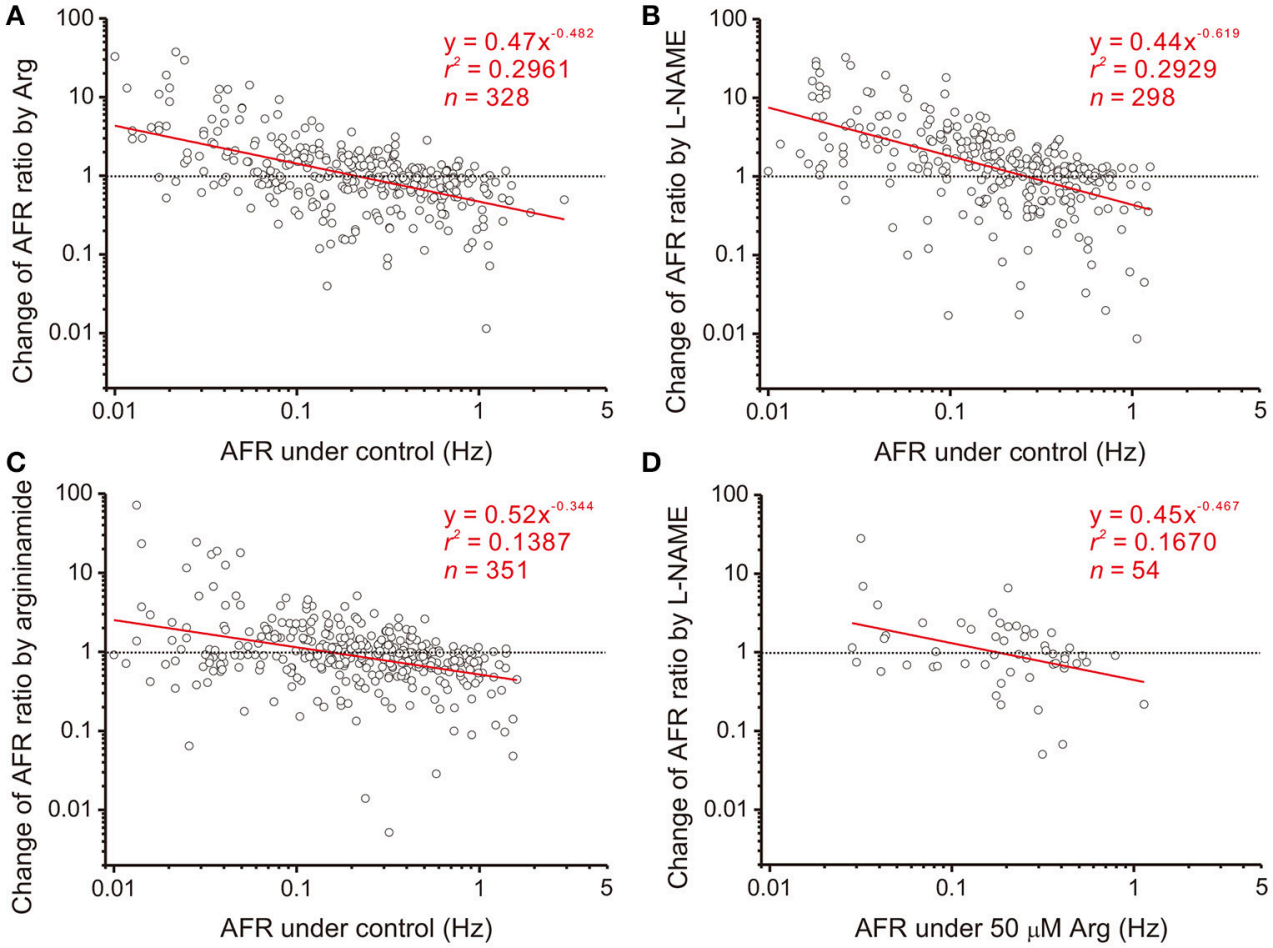
Heterogeneity in NO-mediated changes of firing activities is a power function of their basal activities. Each circle in the plots represents a drug-induced single-fiber response. AFR ratios, y, are the AFR after drug applications divided by the AFR prior to drug applications (i.e., x). Data were obtained from applications of 50 μM Arg **(A)**, 100 μM L-NAME **(B)**, 50 μM L-argininamide (an Arg derivative, **C**) and addition of 100 μM L-NAME with pretreatments of 50 μM Arg **(D)**. All the tests yield a linear data distribution in log-log plots. Data points located near the dashed lines, y = 1, are those responses that applications of drugs exert little effect. As examples in Figure 2 show, the fibers of little drug effects have basal activities as predicted by the regression lines crossing with the dashed lines in Hz: 0.21 **(A)**, 0.26 **(B)**, 0.15 **(C)**, and 0.18 **(D)**. Significance of regression: *P* < 0.00001 in **(A–C)** and *P* < 0.005 in **(D)**. Confidence intervals of the slope (CI_*b*_): **(A)** −0.575 to −0.389, **(B)** −0.745 to −0.493, **(C)** −0.448 to −0.241, and **(D)** −0.802 to −0.133.

Splanchnic sympathetic fiber activities originated from the neonatal rat spinal cord were recorded and NO effects on their spontaneous firing behaviors were examined. Endogenous NOS activities in the spinal cord were enhanced and reduced by bath applications of Arg and L-NAME, respectively. Figure 2 shows the responses of 6 sympathetic single-fibers and demonstrates that either enhancement or reduction of NOS increases, decreases or does not apparently change their spontaneous firing. Analyses were based on the recorded fibers of average firing rates (AFR) ≥ 0.01 Hz. Changes of AFR ratios ≥ 10% were considered responsive to drug applications. In 19 experiments that Arg was applied, 328 single-fibers were recorded. Among them, 138 fiber activities increased, 148 decreased, and 42 did not change. In 17 experiments that L-NAME was applied, 298 single-fibers were recorded. Among them, 184 fiber activities increased, 88 decreased, and 26 did not change. Also, as the examples in Figure 2 demonstrated, fibers of lower spontaneous activities were excited and those of higher activities inhibited by Arg or L-NAME applications. We therefore determined if the heterogeneity in firing responses could be explained by differences in spontaneous activity levels. Drug-induced changes in AFR ratio were plotted against the AFR under control or the AFR after a drug pretreatment (Figure 3). In log-log plots, the data obtained from Arg or L-NAME applications were scattered similarly; the AFR ratios were inversely and linearly correlated with control AFR. The data were successfully regressed by the power functions (Figures 3A,B) and not as well regressed by the logarithmic or the exponential functions (Supplementary Figure 3). To confirm that exogenous applications of Arg specifically promoted NO production and subsequently caused the power-law firing modulations (plFM), an Arg derivative, L-argininamide, was applied for comparisons. The data of Arg applications as shown in Figure 3A were scattered in a pattern similar to the data of L-argininamide applications as shown in Figure 3C, though the latter were fitted with a power function of less negative slope. The slope difference was verified by adding the interaction *G* × *AFR* as a regressor and was found to be significant (*P* < 0.05). Thus, it confirmed an expected difference in the efficacy of NO production between Arg and L-argininamide. By contrast, the L-NAME-induced plFM under experimental conditions with and without exogenous Arg were not different (slope comparisons between regression lines of Figures 3B,D *P* = 0.297), suggesting that exogenous Arg was not critical for NO synthesis under control experimental conditions. The observations that similar plFM are incurred by Arg and L-NAME indicate a paradoxical role of NO in heterogeneous modulation of the population firing behavior.

To further elucidate the paradoxical roles of NO effects in changing spontaneous firing, we examined the data obtained from four experiments that Arg applications were followed by L-NAME applications. There were 40 fibers with activities responsive to both Arg and L-NAME applications. Among them, antagonistic effects elicited by Arg and L-NAME applications were observed in 21 fibers; 13 fibers of control AFR 0.19 ± 0.03 Hz had firing increased by Arg and decreased by L-NAME while 8 fibers of control AFR 0.37 ± 0.10 Hz had firing decreased by Arg and increased by L-NAME. In contrast, paradoxical effects elicited by Arg and L-NAME applications were observed in 19 fibers; 9 fibers of control AFR 0.12 ± 0.03 Hz had firing increased by Arg and L-NAME while 10 fibers of control AFR 0.42 ± 0.14 Hz decreased by both Arg and L-NAME. Thus, the paradoxical roles elicited by perturbation of NOS activities in spontaneous firing were found in 48% of the recorded fibers.

### Involvement of endogenous synaptic activity in NO modulation of firing behaviors

**Figure 4:**
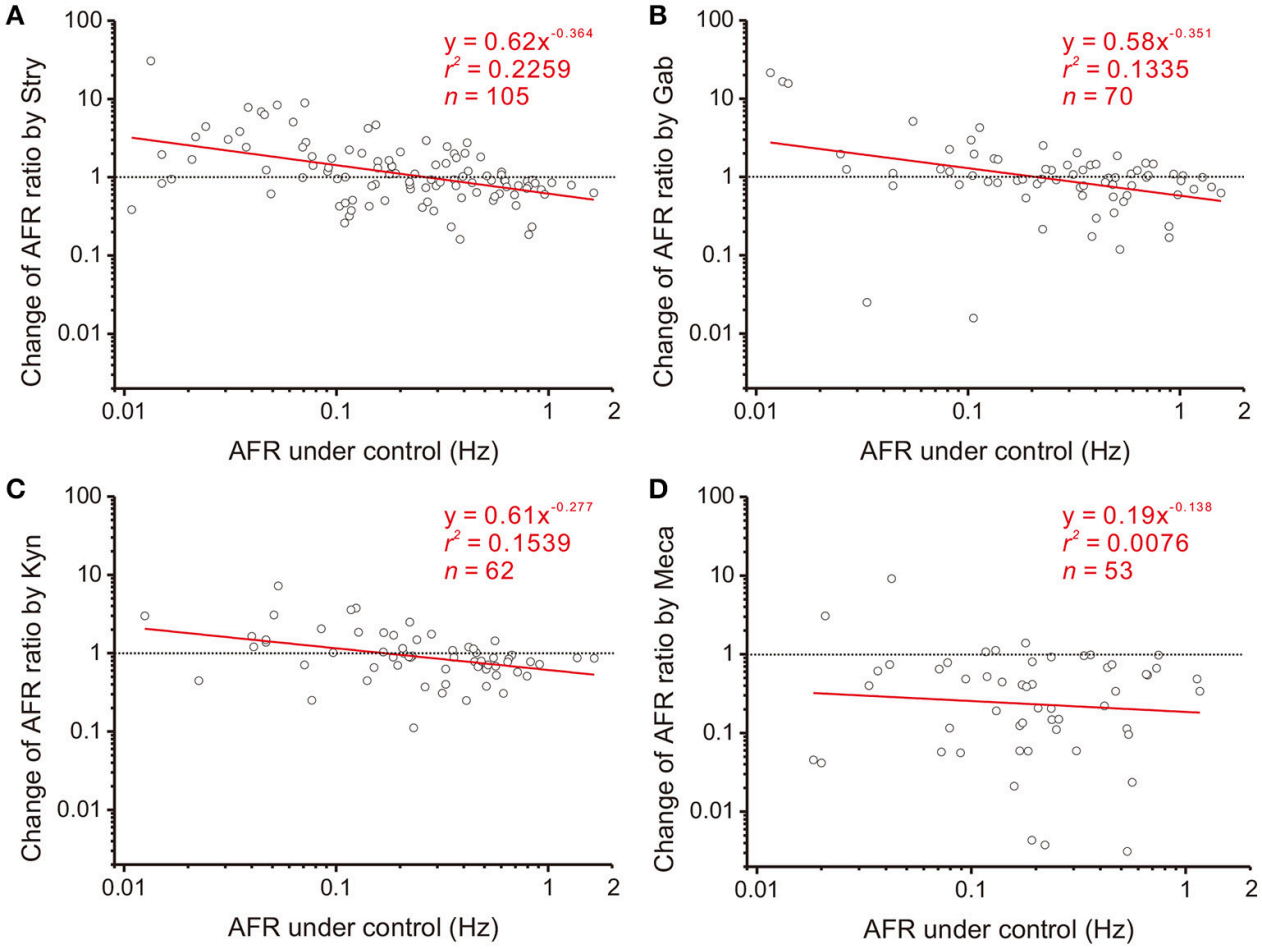
Involvement of endogenous receptor activities in power-law firing modulation (plFM). The log-log plots show the data obtained from bath applications of 2 μM strychnine (Stry, a glycine receptor blocker; **A**), 20 μM gabazine (Gab, a GABA_A_ receptor blocker; **B**), 400 μM kynurenate (Kyn, a broad-spectrum ionotropic glutamate receptor blocker; **C**), and 1 μM mecamylamine (Meca, a nicotinic receptor blocker; **D**). Applications of Stry, Gab, or Kyn caused the plFM. Applications of Meca largely reduced AFR and did not cause the plFM. Significance of regression: *P* < 0.00001 in **(A)**, *P* < 0.005 in **(B,C)** and *P* = 0.5389 in **(D)**. CI_*b*_: **(A)** −0.515 to −0.213, **(B)** −0.600 to −0.102, **(C)** −0.470 to −0.084, and **(D)** −0.648 to 0.371.

**Figure 5:**
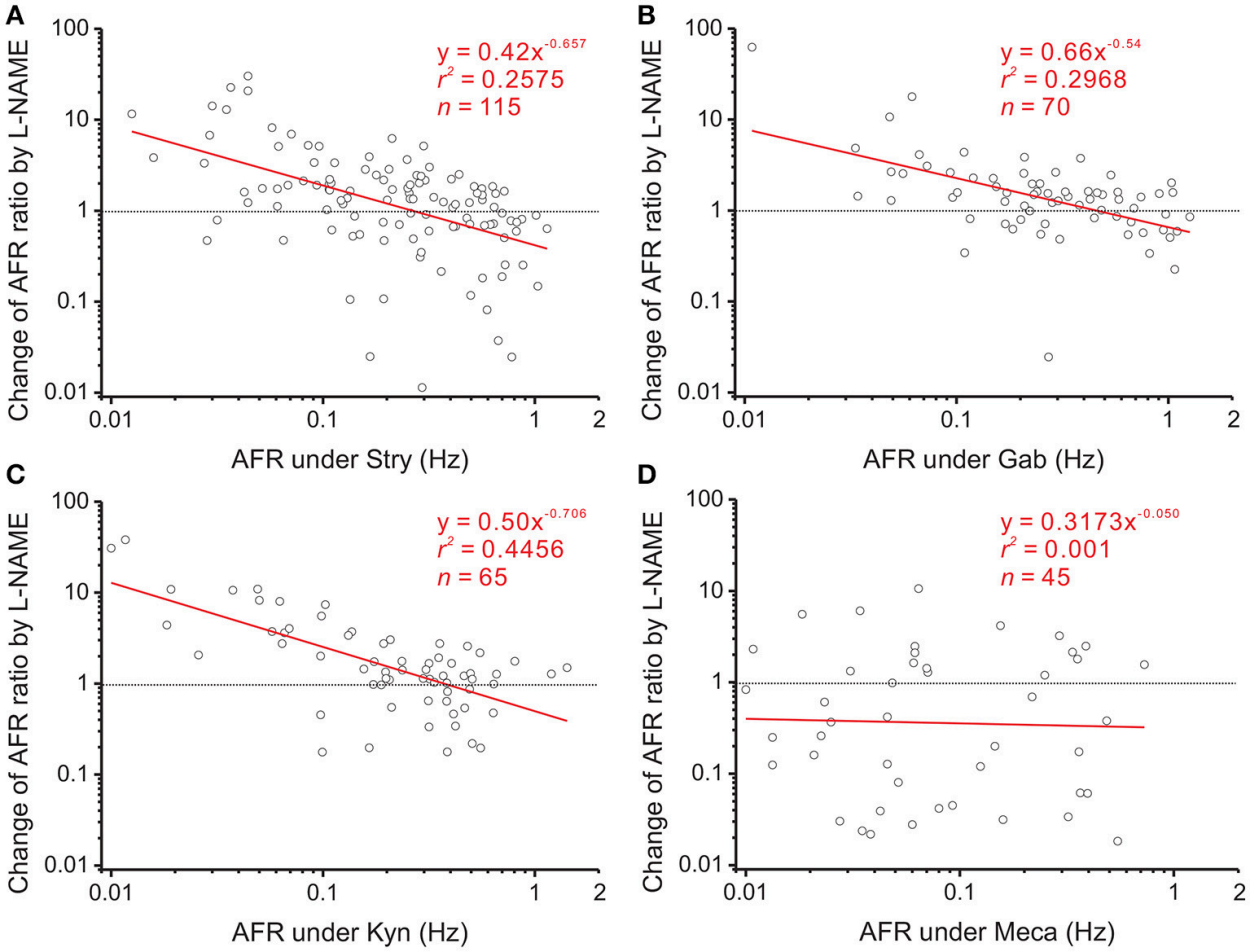
Effects of blocking various endogenous receptor activities on NO-mediated plFM. The log-log plots show the data obtained from bath applications of 100 μM L-NAME after a pretreatment of 2 μM Stry **(A)**, 20 μM Gab **(B)**, 400 μM Kyn **(C)**, or 1 μM Meca **(D)**. Effects of these pretreatments on firing activities were shown in Figure 4. The plFM induced by L-NAME persisted with the pretreatment of Stry, Gab or Kyn. With the pretreatment of Meca, applications of L-NAME did not cause the plFM. Significance of regression: *P* < 0.00001 in **(A–C)** and *P* = 0.8385 in **(D)**. CI_*b*_: **(A)** −0.896 to −0.419, **(B)** −0.771 to −0.309, **(C)** −0.934 to −0.478, and **(D)** −0.594 to 0.495.

NO is a diffusible gas and affects multiple synaptic events. To test the hypothesis that NO-mediated plFM were attributed to NO effects on the synaptic events antecedent to SPNs, endogenous neurotransmissions were first interrupted by applications of various antagonists followed by applications of L-NAME. The data were compared with those acquired from L-NAME applications without antagonist pretreatments, as shown in Figure 3B. On one hand, applications of Stry, Gab, or Kyn caused plFM (Figures 4A–C). With these antagonist pretreatments, similar plFM were elicited by addition of L-NAME (Figures 5A–C). On the other hand, application of Meca did not cause plFM (Figure 4D). With Meca pretreatments, plFM were not elicited by additions of L-NAME (Figure 5D). The slope comparisons between the regression lines obtained from the L-NAME applications with Meca pretreatments and those without were significant (cf. Figures 3B, 5D *P* < 0.002). Thus, NO-mediated plFM required an intact cholinergic neurotransmission working via nicotinic receptors, but not those via glycine, GABA_A_, and ionotropic glutamate receptor activities.

**Figure 6:**
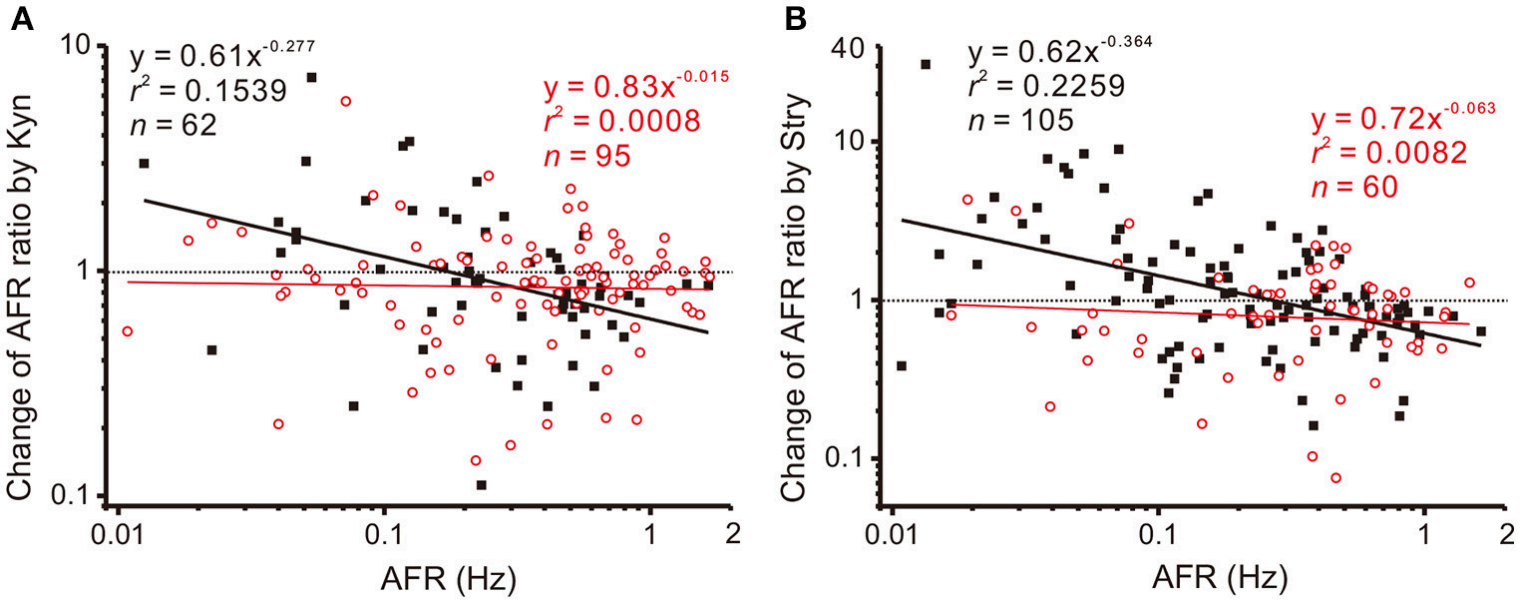
L-NAME pretreatments eliminate Kyn- or Stry-induced plFM. For comparisons, the data in Figure 4 obtained from Kyn or Stry applications without L-NAME pretreatment were replotted here, shown as the solid black squares and the thick black regressed line. Empty red circles and the thin red regression line show the data with L-NAME pretreatments. The data with L-NAME pretreatment were not successfully regressed by the power functions (*P* = 0.7868 and 0.4952 in **A,B**, respectively). Slopes of the regression lines derived from the data with and without L-NAME pretreatments were significantly different (*P* < 0.01 in both **A,B**). CI_*b*_ with L-NAME pretreatments: **(A)** −0.138 to 0.109 and **(B)** −0.272 to 0.147.

The observations that applications of Stry, Gab, or Kyn alter firing in a manner similar to NO-mediated plFM suggest an interplay between NO and glycinergic, GABAergic, or glutamatergic neurotransmission. To test if NO could grade the neurotransmitter activities that participated in plFM, a pretreatment of L-NAME was used to reduce endogenous NO production followed by applications of Stry, Gab, or Kyn. The data were compared with those without L-NAME pretreatments. In the presence of 100 μM L-NAME, the data obtained from applications of 20 μM Gab were successfully regressed by a power function (*y* = 0.91*x*^−0.204^, *r*^2^ = 0.0552, *n* = 104, *P* < 0.05, CI_*b*_: −0.406 to −0.003), which was not significantly different from the one without L-NAME pretreatment (cf. Figure 4B, *P* = 0.5296). However, L-NAME pretreatment eliminated Kyn- or Stry-induced plFM (Figure 6). Thus, a graded glutamatergic or glycinergic neurotransmission that contributes to plFM is secondary to endogenous NO synthesis.

### Enhancement of endogenous cholinergic neurotransmission or exogenous activation of nicotinic receptors modulates firing in power-law manners

**Figure 7:**
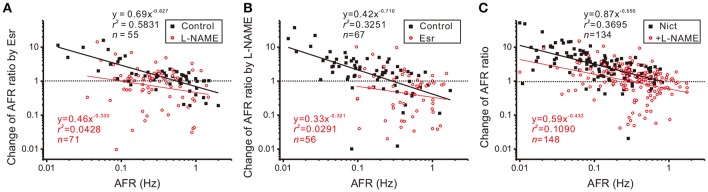
Interplay of cholinergic neurotransmission and NO signaling via nicotinic receptor activation causes plFM. **(A)** Firing modulations induced by applications of 10 μM eserine (Esr, an acetylcholinesterase inhibitor) with or without 100 μM L-NAME pretreatments. Esr-induced plFM were diminished by L-NAME pretreatments. Significance of regression: *P* < 0.00001 under control and *P* = 0.0857 with L-NAME pretreatment. CI_*b*_: −0.794 to −0.459 under control and −0.766 to 0.101 with L-NAME pretreatment. **(B)** Firing modulations induced by applications of 100 μM L-NAME with or without 10 μM Esr pretreatments. L-NAME-induced plFM were diminished by Esr pretreatments. Significance of regression: *P* < 0.00001 under control and *P* = 0.2131 with Esr pretreatments. CI_*b*_: −1.002 to −0.419 under control and −0.902 to 0.260 with Esr pretreatment. **(C)** Firing modulations induced by applications of 1 μM nicotine (Nict, a nicotinic receptor agonist) followed by addition of 100 μM L-NAME. The plFM were induced by Nict and persisted after addition of L-NAME. Significance of regression: *P* < 0.00001 by Nict and *P* < 0.00005 by addition of L-NAME. CI_*b*_: −0.698 to −0.412 by Nict and −0.664 to −0.201 by addition of L-NAME.

The observation that intact nicotinic receptor activities are required for NO-mediated plFM suggests that the cholinergic neurotransmission is upstream to NOS activities. To further clarify the roles of endogenous cholinergic neurotransmission in the plFM, 10 μM Esr was applied to reduce acetylcholinesterase activity and enhance cholinergic neurotransmission. Changes in firing activities often occurred ~5 min after eserine applications. Thus, Esr effects on AFR were evaluated by an epoch of 10 min starting from 5-min after Esr applications. Applications of Esr induced a heterogeneous firing response. A log-log plot of Esr-induced changes in AFR ratios against their control AFR showed a linear data distribution; the data were successfully regressed by a power function (Figure 7A). In the presence of Esr, addition of L-NAME failed to induce the plFM (Figure 7B). In another series of experiments, 100 μM L-NAME was applied as a pretreatment. Esr-induced plFM were diminished (Figure 7A). These results suggest that intact cholinergic neurotransmission and NOS signaling are both required and mutual dependent for plFM.

The involvement of nicotinic receptors in plFM was further investigated. Applications of 1 μM Nict mostly increased spontaneous firing. The log-log plot of Nict-induced change of AFR ratios against control AFR showed a linear data distribution; the data was successfully regressed by a power function (Figure 7C). Addition of L-NAME in the presence of Nict caused plFM, confirming the persistence of NO-mediated plFM when endogenous nicotinic receptor-mediated activities were intact.

### Approximation of plFM by a simple arithmetic model: firing changes in a manner of step increments and fractional reductions

**Figure 8:**
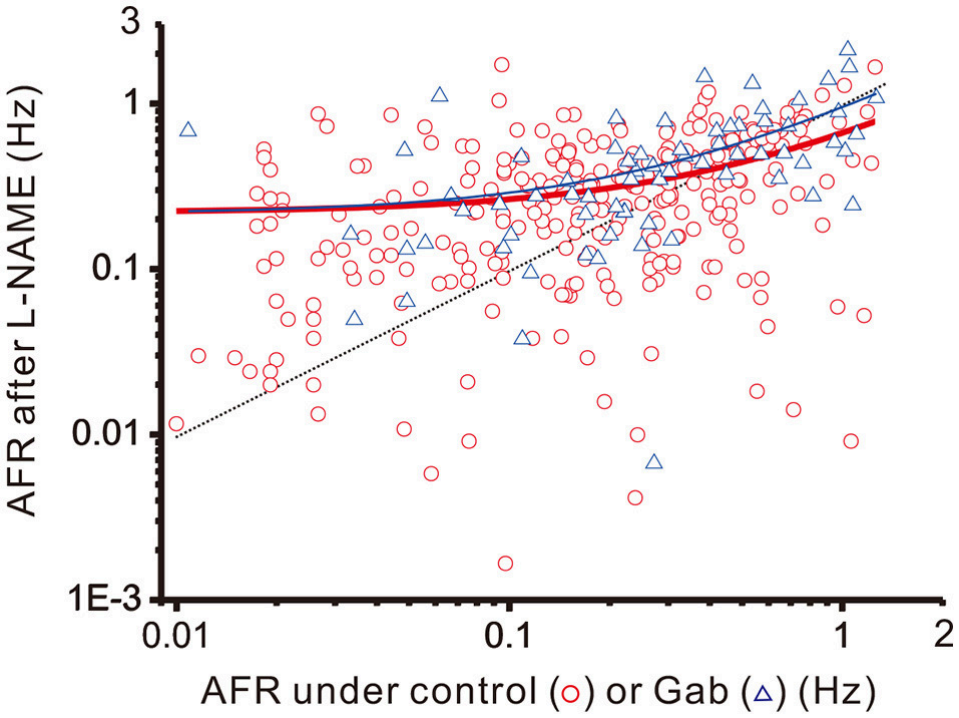
Linear curve fitting for the data obtained from plotting the AFR after L-NAME applications against the AFR prior to L-NAME applications. The plot shows two series of data obtained from the experimental conditions with (blue triangles) or without (red circles) 20 μM Gab pretreatments. Data were fitted by a simple arithmetic model as described in section Materials and Methods to determine the coefficient of step increment (*c*_*s*_) and the coefficient of fractional reduction (*c*_*f*_), as summarized in Table 1. The red thick line depicts the curve fitted for the data without Gab pretreatments (*r*^2^ = 0.1571, *n* = 298, *P* < 0.00001) and the blue thin line depicts that with Gab pretreatments (*r*^2^ = 0.3292, *n* = 70, *P* < 0.00001). The data distributed near the black dashed line were the data not responsive to L-NAME applications. With Gab pretreatments, there was a significant drop of *c*_*f*_ (*P* < 0.05; see Table 1), leading to a slope increase of the fitted curve.

To recapitulate the physiological contexts of plFM, we considered two arithmetic components that commonly altered firing behaviors. One is a step increment of firing, the other a fractional reduction of firing. The latter component is proportional to the original firing activities. The combined effects of these arithmetic components rendered a power-law-like mathematical feature in constructing a plot of AFR ratios against their original AFR (Supplementary Figure 2). The arithmetic components pertinent to the data of L-NAME applications with and without antagonistic pretreatments were compared to elucidate the computational roles of various neurotransmitter activities in generating the NO-mediated plFM. As described in section Materials and Methods, scattered data in the plots of the AFR after L-NAME applications (*x*_*t*_) against their original AFR (*x*_*o*_) were fitted by the equation: *x*_*t*_ = *c*_*s*_ + (1 − *c*_*f*_) • *x*_*o*_ to determine the coefficient of step increments (*c*_*s*_) and the coefficient of fractional reduction (*c*_*f*_). Figure 8 shows an example of the curve fitting for the data of L-NAME applications with and without Gab pretreatments. Table 1 summarizes the extracted *c*_*s*_ and *c*_*f*_ under experimental conditions of various antagonistic pretreatments. Analyses indicated that the *c*_*s*_ and *c*_*f*_ obtained from the data of L-NAME applications were not significantly altered by Stry pretreatments. Both *c*_*s*_ and *c*_*f*_ were reduced by Kyn pretreatments. Only *c*_*f*_ and not *c*_*s*_ was reduced by Gab pretreatments. Thus, endogenous NO by affecting the neurotransmission mediated by ionotropic glutamate receptors could exert contradictory effects on population firing behaviors, i.e., accelerating the firing by boosting the activity levels (i.e., an effect of *c*_*s*_) yet attenuating the firing by augmenting a fractional reduction of their activity levels (i.e., an effect of *c*_*f*_). NO also affected GABAergic neurotransmission via GABA_A_ receptors, which mainly incurred a fractional reduction of population firing and thus contributed to the intercept and not to the slope of a power function.

**Table 1:** Step increment and fractional reduction of sympathetic fiber activities induced by applications of L-NAME with or without antagonist pretreatments.

**Pretreatment**	**C_s_**	**C_f_**	**df**	***P*_Cs_**	***P*_Cf_**
None	0.220 ± 0.022	0.553 ± 0.060	296	–	–
Kyn	0.125 ± 0.052	−0.027 ± 0.131	63	0.036	0.0000194
Stry	0.278 ± 0.048	0.615 ± 0.115	113	0.114	0.295
Gab	0.215 ± 0.062	0.265 ± 0.124	68	0.498	0.0116

C_s_, coefficient of step increment in firing; C_f_, coefficient of fractional reduction in firing; df, degree of freedom. P-values indicate significance of coefficient comparisons with “None.”

## Discussion

Using the neonatal rat spinal cord *in vitro* as an experimental model, this study elucidates the roles of NOS in fine-tuning sympathetic firing behaviors. Manipulations of NOS activities heterogeneously influence the firing activities of different sympathetic fibers, a phenomenon that is related to their basal activities and is best described by a power function. This NO-mediated power-law modulation of sympathetic firing behaviors requires endogenously active nicotinic receptor activities and involves endogenously active glycine, GABA_A_, and ionotropic glutamate receptor activities. Thus, as primed by yet unknown sources of cholinergic neurotransmission, NOS could guard incoming neurotransmitter activities and autonomously grade sympathetic activity levels. We attempted to explain how NO-mediated plFM could occur.

### SPNs are heterogeneous in their activity levels

**Figure 9:**
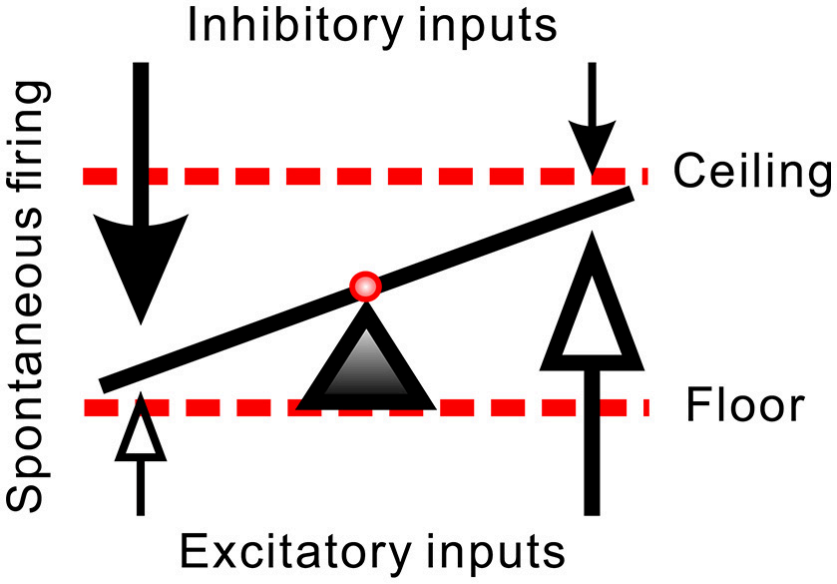
Hypothetical synaptic inputs governing spontaneous firing activities of SPNs. Arrows represent vectors of synaptic inputs. The triad pointing to the slanting line marks the activity level of an SPN. The activity levels result from different extents of inhibitory and excitatory inputs. Effects of a diffusive NO-mediated synaptic modulation on the activity levels are limited by a floor effect of inhibitory inputs (i.e., quiescent SPN cannot be further silenced by excessive inhibition) and by a ceiling effect of excitatory inputs (i.e., active SPN cannot be further activated by excessive excitation). In case that L-NAME enhances synaptic transmissions, the inhibitory floor and excitatory ceiling effects favor a more effective excitatory enhancement in SPNs of low spontaneous activity and more inhibitory reduction in SPNs of high spontaneous activity (i.e., the values of the vectors of low magnitudes in both ends are effectively increased). In case that Arg reduces synaptic transmissions, the same effects favor a removal of strong inhibitory inputs in SPNs of low spontaneous activity and a removal of strong excitatory inputs in SPNs of high spontaneous activity (i.e., the values of the vector of high magnitudes are substantially decreased).

SPNs are heterogeneous. They differ in somatic shapes and sizes and their dendritic projections (Forehand, 1990; Li et al., 1993). Distinct biophysical and histological features endow SPNs with different extents of spontaneous firing activities (Su et al., 2007). SPNs receive direct GABAergic, glycinergic, and glutamatergic inputs (Llewellyn-Smith et al., 1992; Spanswick et al., 1994; Krupp et al., 1997; Su, 2001). By integrating different extents of excitatory and inhibitory inputs, SPNs differ in their spontaneous firing activities. Under current experimental conditions, their AFR range from 0 to ~2 Hz. This observation implies that some SPNs are predominantly inhibited while some relatively excited. A setting point of their firing capacity is then determined by the extents of inhibition and excitation, as illustrated in Figure 9. On one hand, the observation that Gab- or Stry-induced excitation in those fibers of low spontaneous activities may be explained by a disinhibitory effect. However, this disinhibitory hypothesis fails to explain why applications of Gab or Stry tend to suppress firing in those fibers of high spontaneous activities. On the other hand, the observation that applications of Kyn reduce firing in fibers of high spontaneous activities may be explained by a direct excitatory glutamatergic inputs to the somatodendritic regions of these fibers. Again, this excitatory hypothesis fails to explain why Kyn applications tend to excite those fibers of low spontaneous activities. While an indirect path, constructed by a glutamatergic activation of GABAergic neurotransmissions that subsequently inhibit glycinergic inhibition, could explain Kyn-induced excitation in fibers of low spontaneous activities, this speculation was not supported by available evidence. Thus, while a balance of excitatory and inhibitory inputs to SPNs can justify the level of spontaneous firing activities, the other variables should be introduced to explain the antagonist-induced changes of firing in a power-law manner.

### NO affects various channel activities incurring multifarious postsynaptic and presynaptic effects

The paradoxical effects on firing modulation incurred by Arg and L-NAME as observed in this study need to be reconciled. NO exert multifarious effects on neuronal activities via both pre- and post-synaptic events. NO effects on presynaptic terminals may enhance glutamate or GABA release in the hippocampal neurons (Zanelli et al., 2009; Neitz et al., 2011), potentiates GABAergic neurotransmissions in the striatal spiny projecting neurons (Sagi et al., 2014) and enhances acetylcholine release in the pontine reticular formation (Leonard and Lydic, 1997). In lines with enhanced presynaptic events, NO effects on postsynaptic membranes reduces outwardly rectified K^+^ channels in type I hair cells in semicircular canals, increases their whole-cell membrane input resistance and augments the receptor potentials (Chen and Eatock, 2000). A boost of synaptic efficacy by NO, however, could be counteracted by a down-regulation of the postsynaptic excitability. For instance, NO reduces AMPA or NMDA receptor-mediated excitatory postsynaptic potentials in the principal neurons of mouse median nucleus of trapezoid body (Steinert et al., 2008). Also, activity-dependent induction of NO synthesis suppresses Kv3 and potentiates Kv2 in the neurons of the auditory brainstem and the hippocampus, which serves as a mechanism to transform synaptic integration and adjust synaptic strength for homeostatic regulation (Steinert et al., 2011). Likewise, NO has been shown to sustain low-conductance K^+^ channel activities in the basolateral membrane of the cortical collecting duct of the rat kidney (Lu and Wang, 1996) and activate K_ATP_ channels in HEK 293 cells (Lin et al., 2004). Moreover, cell-specific opposite effects on the intrinsic excitability have been reported in snail B5 and B19 neurons, where self-produced NO excites B5 neurons and inhibits B19 neurons (Zhong et al., 2015). The counteracting effects of NO on pre- and post-synaptic events in affecting neuronal excitability partly explain why either increasing or decreasing NO production by exogenous applications of Arg or L-NAME yields a similar manner in altering the sympathetic fiber activities.

**Figure 10:**
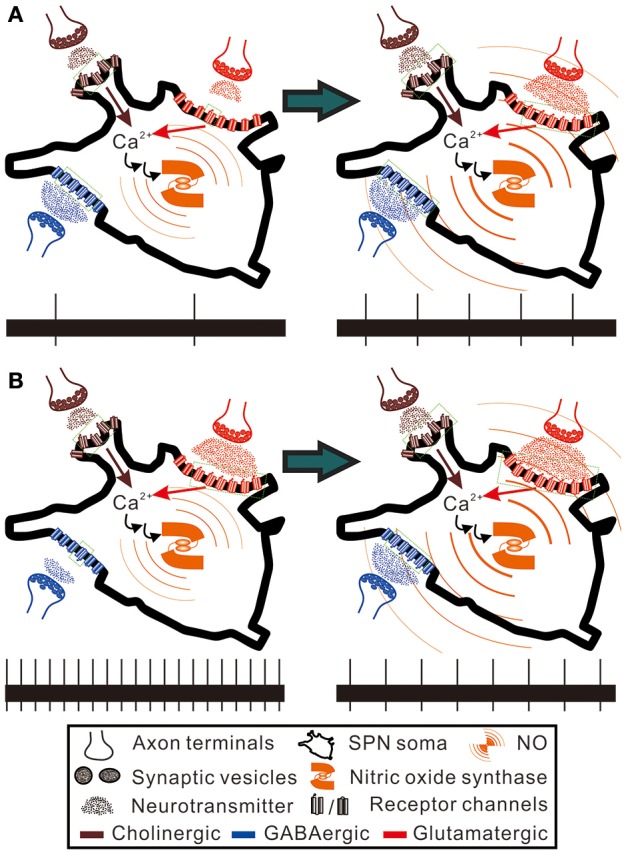
Hypothetical scenarios to explain the paradoxical spiking responses elicited by NO enhancement in SPNs of low and high spontaneous activities. Here, the SPNs of high and low activity levels are presumed to express nitric oxide synthase and are bombarded by GABAergic and glutamatergic synaptic inputs to different extents, as hypothesized in Figure 9. Also, NO is presumed to enhance cholinergic, GABAergic and glutamatergic neurotransmission to SPNs (see section Discussion for references). Brown and red arrows indicate Ca^2+^ influxes from nicotinic cholinergic receptors and ionotropic glutamate receptors, which participate in activating nitric oxide synthase. Green dotted squares enclose the receptor channels in open states. The bottom trace in each panel shows a 20-s epoch of spiking activities under control conditions (the left panels) and during NO enhancement (the right panels). **(A)** NO enhancement increases spiking activities from 0.1 to 0.25 Hz. **(B)** NO enhancement decreases spiking activities from 1 to 0.4 Hz. By counting the numbers of the receptor channels in open and closed states, NO enhancement causes only a fractional change of GABAergic neurotransmission in **(A)** and a fractional change of glutamatergic neurotransmission in **(B)**. In contrast, NO enhancement causes a five-fold increase of glutamatergic neurotransmission in **(A)** and a three-fold increase of GABAergic neurotransmission in **(B)**. Thus, alteration of the spiking activities by NO predominantly follows a recruitment of closed receptor channels or silent synapses. The hypothetical scenarios explain an activity-dependent heterogeneity in NO modulation of sympathetic firing behaviors.

In addition to the disparate pre- and postsynaptic effects on firing activities, NO effects on synaptic strength yield an activity-dependent ceiling or floor effect. NO produced from immature rat SPNs may act as a retrograde messenger to potentiate both excitatory and inhibitory synaptic inputs to themselves (Wu and Dun, 1996; Wu et al., 1997). On the assumptions that the spontaneous activity levels of SPNs reflect an integration of different extents of inhibitory and excitatory synaptic inputs, a reduced presynaptic inhibition would excite more in the SPNs with lower spontaneous activities, while a reduced presynaptic excitation would suppress more in the SPNs with higher spontaneous activities. On the other hand, an enhancement of neurotransmission could strengthen weak synaptic activities, leading to a predominant enhancement of the weak excitation in slow-firing fibers and the weak inhibition in fibers of high spontaneous activities. Thus, as illustrated in Figure 9, the paradoxical effects resulted from applications of Arg and L-NAME eliciting similar firing responses could then be explained by a floor effect on inhibition, which cannot further decrease firing in an already strong inhibitory synaptic transmission and a ceiling effect on excitation, which cannot further increase firing in an already strong excitatory synaptic transmission. On the premise of hypothetical scenarios, Figure 10 further illustrates how the floor and the ceiling effects in limiting the change in synaptic transmissions lead to the NO-induced paradoxical effects on the spiking responses of the SPNs with either low or high spontaneous activities. By boosting and suppressing the spiking activities in the SPNs with low and high spontaneous activities, respectively, NO modulation is activity-dependent and serves the best for a homeostatic regulation of SPN activity levels.

Different populations of SPNs that innervate different sympathetic ganglions differ in the percentages of the neurons that express NOS (Hinrichs and Llewellyn-Smith, 2009). This observation intriguingly implies that SPNs targeting at different organs are differentially affected by different amounts of NO. Indeed, low and high concentrations of NO differentially augment and reduce delayed-rectified K^+^ channel currents (Han et al., 2006). Thus, different amounts of NO produced in SPNs targeting at different organs may alter sympathetic firing behaviors heterogeneously, as we have observed and serves as a neural mechanism for differential controls of various sympathetic targeting organs.

Although not without debate, the sympathetic outflows supplying the cardiovascular systems are predominantly reduced by NO production in the spinal cord, as inferred by observing changes in systemic arterial blood pressure (Iida, 1999; Arnolda et al., 2000; Sabino et al., 2011). Consistent with this view, intrathecal L-NAME increases blood pressure in both conscious rats (Lu et al., 1999) and anesthetized rats (Koga et al., 1999). However, conflicting evidence has also been reported. Intrathecal sodium nitroprusside (an NO donor) or L-NAME cause an increase or a decrease of blood pressure in anesthetized rats (Lee et al., 1996). Moreover, intrathecal Arg maintains mean blood pressure during hemorrhage in anesthetized rats (Malik et al., 2007). While these findings support a hypothetical NO-mediated activation of vasomotor tone in the spinal cord, a recent study demonstrates that NO produced by NOS-I in SPNs contributes to maintain while NO produced by NOS-II in the vicinity of the intermediate lateral cell column tonically depress mean arterial blood pressure (Poon et al., 2016). By direct neural recordings, it has been demonstrated that intrathecal Arg increases renal sympathetic nerve activities in anesthetized rabbits (Hakim et al., 1995). Despite the differences in animal species and experimental conditions, these discrepant observations in NO-mediated regulation of sympathetic outflows in the spinal cord could be reconciled by the current observations that NO-mediated sympathetic control at the spinal cord level is heterogeneous. It should be recalled that the target organs indirectly controlled by the SPNs in this study were not determined. Thus, heterogeneity in sympathetic firing response to NO as presented here may not be relevant or confined to address central NO effects on cardiovascular regulations.

### Nicotinic receptor activities as a primer of NOS-mediated firing modulation

One intriguing finding in this study is that endogenous nicotinic receptor activities are required for NO-mediated plFM. This suggests that NO production is downstream to endogenous cholinergic neurotransmission. Indeed, this view is supported by the finding that acetylcholine stimulates NO release from rat thoracolumbar spinal cord (Xu et al., 1996). The linkage of NO signaling with cholinergic neurotransmission has also been reported in the other systems. For instance, activation of cholinergic neurotransmission in the mouse striatum incurs an NO-dependent short-term plasticity (Blomeley et al., 2015). Also, exogenous activation of nicotinic receptors can protect the heart against ventricular fibrillation via an NO-dependent mechanism (Kalla et al., 2016). Since NO production is Ca^2+^-dependent, the effects of nicotinic receptor activation on Ca^2+^ influx can then be translated into an activity-dependent NO production from those SPNs expressing NOS, thereby serving as a primer of plFM.

The other intriguing finding in this study is a mutual dependence of acetylcholinesterase and NOS activities underlying Esr- or L-NAME-induced plFM. This suggests that a firing modulation in this manner requires intact enzyme activities for degradation of acetylcholine or the production of NO. An interplay between NO and acetylcholinesterase does exist at subcellular levels. Acetylcholinesterase conformational states influence NO mobilization in erythrocytes (Teixeira et al., 2015). Studies using brain homogenates demonstrate that NO can inhibit acetylcholinesterase activities (Udayabanu et al., 2008). If this scenario indeed occurs in the cholinergic synapses laid by some SPNs with intraspinal axon collaterals (Su et al., 2007), NO may indirectly enhance cholinergic neurotransmission and help to sustain a nicotinic receptor-based SND as we have reported (Chen and Su, 2006). Thus, either an inhibition of NOS by L-NAME or a nicotinic receptor desensitization caused by Esr-induced excessive acetylcholine accumulation in the cholinergic synapses may distort NO production and affect the manner of firing modulation.

### The plFM are caused by NO effects on various synaptic activities that change firing in a manner of step increments and fractional reductions

The heterogeneity in firing responses to NO manipulation is well described by the power functions. However, this mathematical feature does not explicitly unravel the physiological contexts of plFM. We therefore attempted to approximate the power function by introducing two arithmetic components and used step increments and fractional reductions to recapitulate the drug-induced changes of firing. We found that NO effects on GABA_A_ receptor activities contributed to a fractional reduction of firing, whilst effects on ionotropic glutamate receptor activities contributed to both arithmetic components. This simple arithmetic model has successfully simulated the plFM and neatly explained the computational features of different neurotransmission underlying the heterogeneous firing responses. However, how NO can alter sympathetic firing in a manner of step increments or fractional reduction still remain elusive. As NO may enhance both excitatory and inhibitory neurotransmitter release and activate postsynaptic membrane K^+^ conductance, it may cause a shunting effect. A shunting effect by lowering the membrane input resistance can produce an incremental or a fractional change of basal firing. This possibility has been elegantly demonstrated by the theoretical works in studying the effects of shunting inhibition on firing rates (Holt and Koch, 1997; Ly and Doiron, 2009). Thus, we envisaged that, by amalgamating various neurotransmitter activities, NO incurred a shunting effect on postsynaptic membrane properties, which contributed to the firing modulation in a power-law manner.

### Significance of heterogeneity in NO modulation of population firing in power-law manners

Sympathetic targets are heterogeneous and may require a differential yet coordinated ensemble commands for dynamic controls. Power-law modulation of population firing via NO signaling in the final gates of the central sympathetic neural circuits provides a simple solution to generate the complex commands for differential controls. By respective down- and up-regulating the activity levels in active and quiescent SPNs, an NO-mediated modulation of population firing in the power-law manners is ideal for homeostatic regulation of SPN activities and protects their innervated target organs from a devastating sympathetic overflow.

## Author contributions

C-KS: designed research, analyzed data, interpreted results, drafted and edited manuscript; Y-YC: performed experiments; C-MH: initiated researches, discussed experimental results, edited manuscript, and approved the final version to be published.

### Conflict of interest statement

The authors declare that the research was conducted in the absence of any commercial or financial relationships that could be construed as a potential conflict of interest.
