# Supplementary material for: Nitric Oxide Orchestrates a Power-Law Modulation of Sympathetic Firing Behaviors in Neonatal Rat Spinal Cords
Source: Front Physiol. 2018 Mar 6;9:163. doi: 10.3389/fphys.2018.00163 (PMC5845561; doi:10.3389/fphys.2018.00163)
Supplement: Supplementary file 1 [file Image1.PDF]

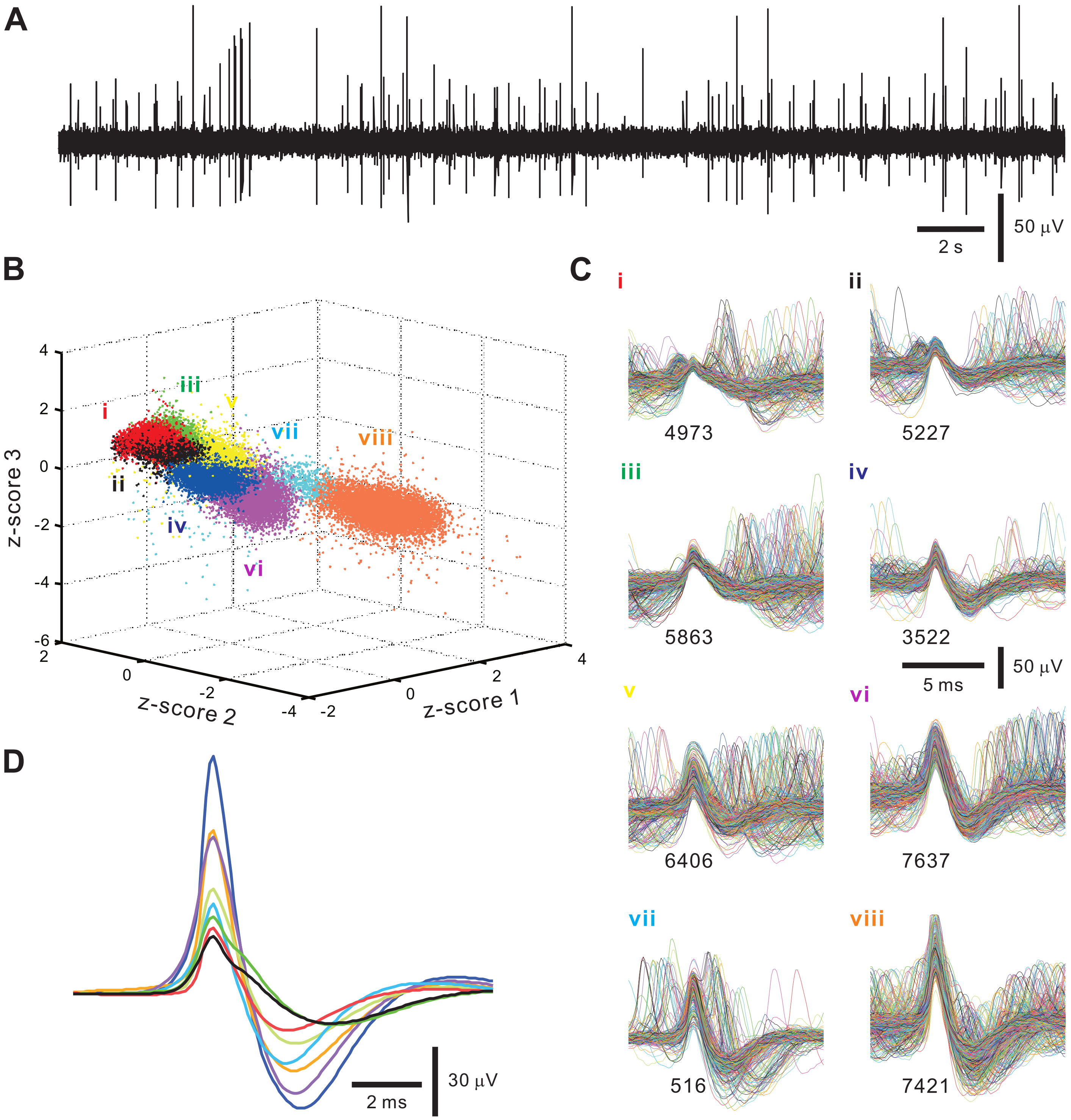

Supplementary Figure 1. Spike sorting of oligofiber activities. **(A)** An original trace of oligofiber activities showing distinct spike potentials. **(B)** 3-D plot of the waveform features of 41565 spikes recorded from a 140-min epoch. Waveform features are expressed in z-scores of peak amplitudes (1), peak roundness (2), and repolarization slopes (3). Data clusters (i–viii, as colors coded) are acquired by *k*-means clustering of the waveform features as previously described (Su et al., Front Comput Neurosci. 2013). **(C)** Superimposed traces showing the spike potentials obtained from 8 data clusters as shown in **(B)**. Numerical values indicate the number of spikes in the clusters. **(D)** Averaged spike waveforms from each cluster in **(C)** were superimposed to demonstrate distinct waveform features of each fiber. Spike waveforms of peak amplitudes from low to high were obtained from the data clusters as labeled i to viii in **(B)** and **(C)**.
