# Supplementary material for: Nitric Oxide Orchestrates a Power-Law Modulation of Sympathetic Firing Behaviors in Neonatal Rat Spinal Cords
Source: Front Physiol. 2018 Mar 6;9:163. doi: 10.3389/fphys.2018.00163 (PMC5845561; doi:10.3389/fphys.2018.00163)
Supplement: Supplementary file 2 [file Image2.PDF]

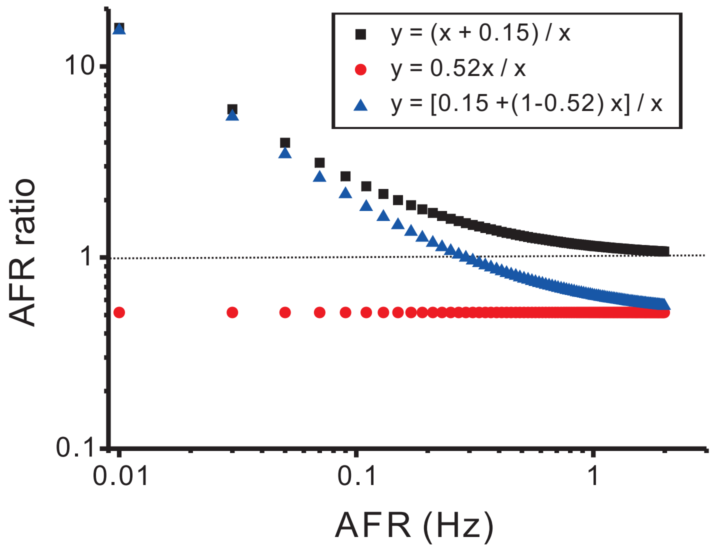

Supplementary Figure 2. Plot of the AFR ratios against the AFR in a log-log scale to recapitulate the arithmetic features of the data distribution in a power-law manner. Simulated data are acquired using the equations as shown. Parametrical values in the equations were acquired by a curve fitting for the data of Arg applications, using the equation as described in METHODS. Black squares are the simulated data assuming a step increment of 0.15 Hz, which have a data distribution pattern similar to a power function. Red circles are the simulated data assuming a fractional reduction of 48% or remaining 52% of their original AFR, which display as a flat line in parallel with x-axis. Blue triangles are the simulated data of 0.15 Hz step increment plus 52% fractional reduction, showing the data distribution in a power-law-like manner. Note the declination of the curve can reaches a level of AFR ratio <1, only when the simulated data combined both arithmetic components.
