# Supplementary material for: Nitric Oxide Orchestrates a Power-Law Modulation of Sympathetic Firing Behaviors in Neonatal Rat Spinal Cords
Source: Front Physiol. 2018 Mar 6;9:163. doi: 10.3389/fphys.2018.00163 (PMC5845561; doi:10.3389/fphys.2018.00163)
Supplement: Supplementary file 3 [file Image3.PDF]

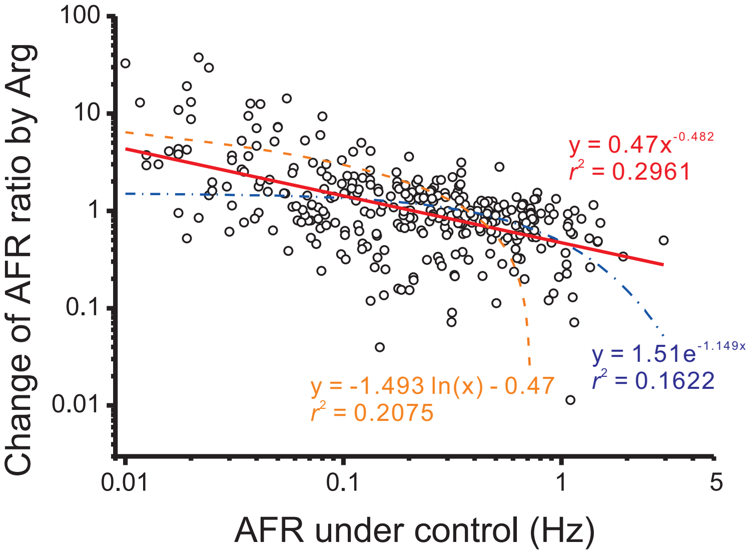

Supplementary Figure 3. Regression by different power-like models. Circles show the data obtained from Arg applications. The data are well regressed by a power function (red thick line), but poorly regressed by a logarithmic function (Mustard yellow dash line) or an exponential function (blue dash-dotted line). The coefficient of determination (r^2) derived from the regression by the power function has the highest value.
